# Virtual and In Vitro Screens Reveal a Potential Pharmacophore that Avoids the Fibrillization of Aβ_1–42_

**DOI:** 10.1371/journal.pone.0130263

**Published:** 2015-07-14

**Authors:** Maricarmen Hernández-Rodríguez, José Correa-Basurto, María Inés Nicolás-Vázquez, René Miranda-Ruvalcaba, Claudia Guadalupe Benítez-Cardoza, Aldo Arturo Reséndiz-Albor, Juan Vicente Méndez-Méndez, Martha C. Rosales-Hernández

**Affiliations:** 1 Laboratorio de Modelado Molecular y Diseño de Fármacos, Escuela Superior de Medicina, Instituto Politécnico Nacional, Plan de San Luis y Díaz Mirón S/N, Delegación Miguel Hidalgo, México D.F., México; 2 Laboratorio de Biofísica y Biocatálisis, Escuela Superior de Medicina, Instituto Politécnico Nacional, Plan de San Luis y Díaz Mirón S/N, Delegación Miguel Hidalgo, México D.F., México; 3 Quimica inorgánica-orgánica del Departamento de Ciencias Químicas, de la Facultad de Estudios Superiores Cuautitlán Campo 1, Universidad Nacional Autónoma de México, Avenida 1o de Mayo S/N, Santa María las Torres, Cuautitlán Izcalli, Estado de México, México; 4 Laboratorio de Investigación Bioquímica, Sección de Estudios de Posgrado e Investigación, Escuela Nacional de Medicina y Homeopatía, Instituto Politécnico Nacional, Guillermo Massieu H 239, Gustavo A. Madero, La Escalera, México D.F., México; 5 Laboratorio de Investigación en Inmunología., Escuela Superior de Medicina, Instituto Politécnico Nacional, Plan de San Luis y Díaz Mirón S/N, Delegación Miguel Hidalgo, México D.F., México; 6 Centro de Nanociencias y Micro y Nanotecnología, Instituto Politécnico Nacional, Luis Enrique Erro S/N, U. Prof Adolfo López Mateos, Gustavo A. Madero, México D.F., México; Consiglio Nazionale delle Ricerche, ITALY

## Abstract

Among the multiple factors that induce Alzheimer’s disease, aggregation of the amyloid β peptide (Aβ) is considered the most important due to the ability of the 42-amino acid Aβ peptides (Aβ_1–42_) to form oligomers and fibrils, which constitute Aβ pathological aggregates. For this reason, the development of inhibitors of Aβ_1–42_ pathological aggregation represents a field of research interest. Several Aβ_1–42_ fibrillization inhibitors possess tertiary amine and aromatic moieties. In the present study, we selected 26 compounds containing tertiary amine and aromatic moieties with or without substituents and performed theoretical studies that allowed us to select four compounds according to their free energy values for Aβ_1–42_ in α-helix (Aβ-α), random coil (Aβ-RC) and β-sheet (Aβ-β) conformations. Docking studies revealed that compound 5 had a higher affinity for Aβ-α and Aβ-RC than the other compounds. *In vitro*, this compound was able to abolish Thioflavin T fluorescence and favored an RC conformation of Aβ_1–42_ in circular dichroism studies, resulting in the formation of amorphous aggregates as shown by atomic force microscopy. The results obtained from quantum studies allowed us to identify a possible pharmacophore that can be used to design Aβ_1–42_ aggregation inhibitors. In conclusion, compounds with higher affinity for Aβ-α and Aβ-RC prevented the formation of oligomeric species.

## Introduction

Alzheimer’s disease (AD) is a progressive neurodegenerative disorder that is characterized by extracellular fibrillary deposits and intracellular neurofibrillary tangles [1]. The primary component of the AD-associated extracellular deposits is a 4-kD peptide that is commonly known as amyloid-β (Aβ) [2]. Aβ originates from the Aβ precursor protein [3], which is hydrolyzed by the β- and γ-secretases to release Aβ peptides containing 39–43 amino acid residues. However, the most important peptide contains 42 residues (Aβ_1–42_) [4]. During fibrillogenesis, Aβ_1–42_ undergoes a conformational change from an α-helix to parallel β-sheets, which are connected by a bent structure encompassing residues 23–29, and the close distance between the side chains of Asp23 and Lys28 forms an electrostatic interaction [5]. This curved structure may be rate-limiting in fibril formation [6]. In recent years, the formation of amyloid fibrils has been shown to be more complex than a linear sequential monomer-to-fibril reaction and consists of several toxic intermediates, including soluble oligomers [7–8], that can bind to hippocampal neurons and induce synaptic plasticity dysfunction [9].

Therefore, many Aβ fibrillogenesis inhibitors that contain aromatic rings and/or amines have been identified through compound library screens and by rational design strategies [10–12]. However, these compounds bind to the elongated β-sheet conformation of Aβ_1–42_ to prevent its polymerization [12–16]. A potential problem with this strategy is that blocking the later stages of fibril formation favors the formation of the prefibrillary oligomeric forms that are even more cytotoxic than the fibrils [10]. Furthermore, the evaluation of pharmacophores that prevent amyloid aggregation has been proposed, but these pharmacophores have only been evaluated during the fibrillization process and, in some cases, only target a segment of Aβ_1–42_ [17–18]. Thus, the design of compounds with greater affinity for the α-helix or random coil (RC) conformations of Aβ_1–42_ than the β-sheet conformation could block the adoption or destabilization of the Aβ_1–42_ β-sheet and could be good oligomerization inhibitors [19–21].

Using *in silico* and *in vitro* studies, our group has demonstrated that electrostatic interactions between the lateral chains of Glu22 and Asp23 in the Aβ_1–42_ turn conformation and a chemical group with a positive charge (such as copper) prevent the formation of the turn, which is necessary during the pathological aggregation of Aβ_1–42_ [22]. In addition, molecular dynamics (MD) simulations have demonstrated that the π–π interaction between the lateral chains of Phe19 and Phe20 favors the α-helix to β-sheet conformational change [21].

In the present study, we focused on small ligands that have tertiary amine and aromatic moieties with or without substituents to identify a possible pharmacophore that could prevent the salt bridge formation and consequently avoid the adoption of the β-sheet conformation of Aβ_1–42_. Twenty-six ligands were chosen from the Sigma–Aldrich database and subjected to docking studies to evaluate their binding affinity and free energy values (ΔG) on three different conformations of Aβ_1–42_ (α-helix, random coil and β-sheet). Based on their affinity toward the Aβ_1–42_ conformations, we selected four compounds (compounds 5, 8, 14 and 19) and used the Thioflavin T (ThT) fluorescence assay to evaluate their *in vitro* activities as Aβ_1–42_ fibrillization inhibitors. The conformation of Aβ_1–42_ in the presence of these compounds was evaluated using circular dichroism (CD) spectroscopy, and the morphology of Aβ_1–42_ was determined using atomic force microscopy (AFM). Once the *in vitro* activity was evaluated, the IC_50_ values of compounds 5 and 8 were determined. Finally, quantum chemistry studies were performed to analyze the electronic behavior and the molecular basis of Aβ_1–42_ recognition by compounds 5 and 8.

## Materials and Methods

**Fig 1:**
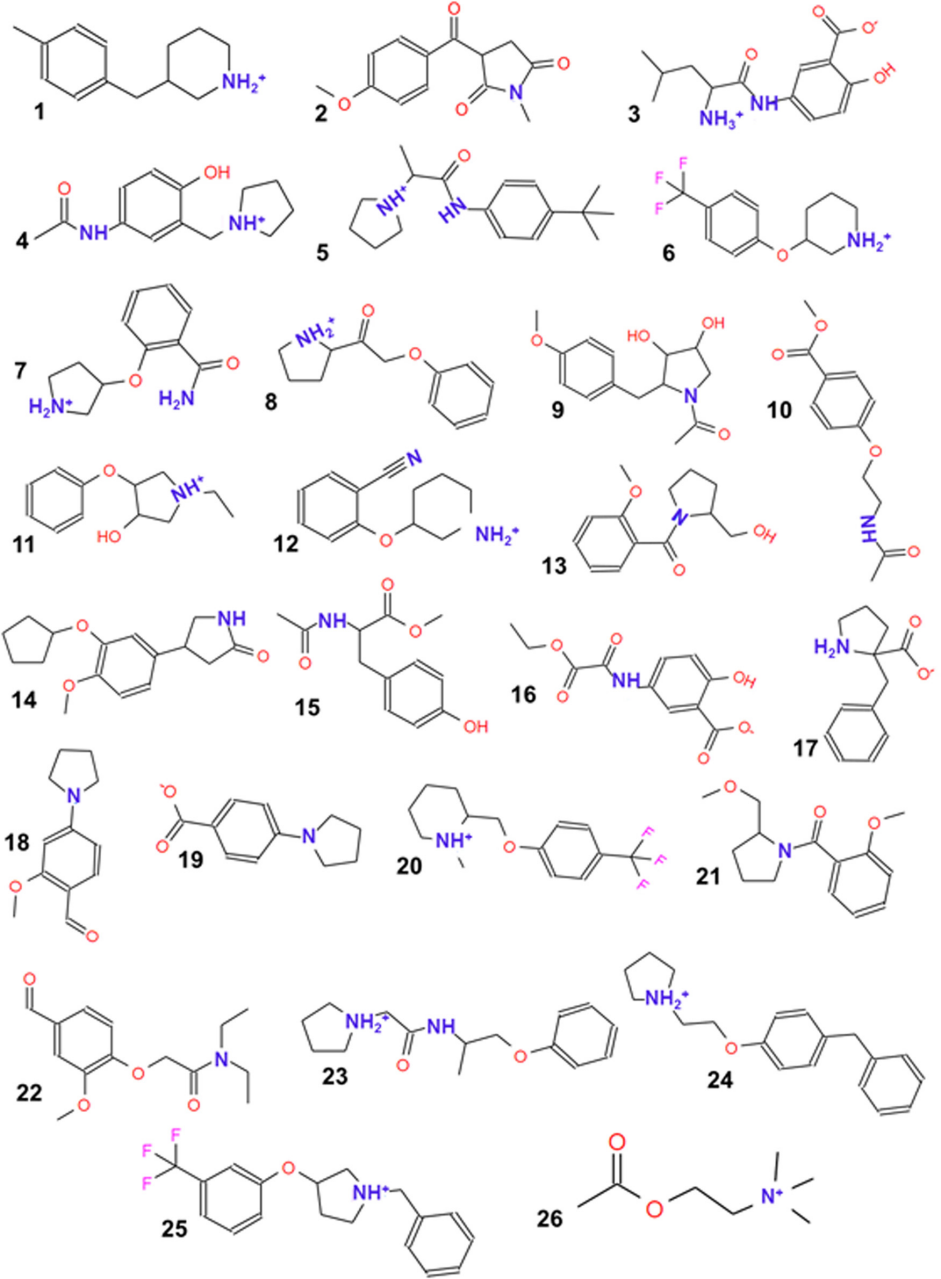
Chemical structures of the selected compounds used as possible Aβ_1–42_ oligomerization inhibitors. All of the compounds selected contained an amine and/or aromatic ring in their structure. However, not all of the compounds could acquire a positive charge at physiological pH. The compounds are shown with their protonation states based on their pKas.

Because most of the ligands that can inhibit Aβ_1–42_ oligomerization possess an amine and an aromatic ring [10–12], 26 ligands (Fig 1) with a molecular weight (MW) of <500 and with amine and aromatic moieties were chosen for docking studies to evaluate their binding to Aβ_1–42_. Among these compounds, we selected acetylcholine (ACh) to evaluate the influence of the lack of an aromatic group in the recognition by Aβ_1–42_ (Fig 1; compound 26). The MarvinSketch server (http://www.chemaxon.com/marvin/sketch/index.jsp) was used to determine the protonation state (pKa) of the compounds according to the ionizable groups at physiological pH (7.35–7.45).

### Ligand selection

Because most of the ligands that can inhibit Aβ_1–42_ oligomerization possess an amine and an aromatic ring [10–12], 26 ligands (Fig 1) with a molecular weight (MW) of <500 and with amine and aromatic moieties were chosen for docking studies to evaluate their binding to Aβ_1–42_. Among these compounds, we selected acetylcholine (ACh) to evaluate the influence of the lack of an aromatic group in the recognition by Aβ_1–42_ (Fig 1; compound 26). The MarvinSketch server (http://www.chemaxon.com/marvin/sketch/index.jsp) was used to determine the protonation state (pKa) of the compounds according to the ionizable groups at physiological pH (7.35–7.45).

### Molecular docking

We selected three Aβ_1–42_ structures in different structural conformations to sample different Aβ_1–42_ conformations. Two of them were obtained from the Protein Data Bank (www.rcsb.org). The first corresponds to Aβ_1–42_ in an α-helix, PDB id: 1Z0Q, and the second corresponds to Aβ_17–42_ in a β-sheet, PDB id: 2BEG. Both structures have been employed in several research studies [22–25]. The structure of the RC conformation corresponds to the Aβ_1–42_ conformer obtained at 10 ns through molecular dynamics (MD) simulations of 1ZOQ, which has been reported by our research group [26]. Additionally, the percentages of secondary structures for each Aβ conformation were calculated using the stride server [27] to provide information on the Aβ_1–42_ structures employed.

To prepare the structures for docking studies, all of the possible rotable bonds and partial atomic charges (Gasteiger-Marsili formalism) of the ligands, as well as the Kollman charges for all of the atoms in the peptide, were assigned using AutoDock Tools version 3.4 [28]. The ligands were docked on the Aβ_1–42_ conformers using AutoDock version 4.2.0 with the hybrid Lamarckian Genetic Algorithm as the search method, an initial population of 100 randomly placed individuals and a maximum of 1.0×10^7^ energy evaluations [29]. The resulting docked orientations that were clustered together occurred within a root mean square deviation of 0.5 Å. The lowest free energy cluster for each docked orientation returned by AutoDock was used for further analysis, and all other parameters were maintained at the default settings [29]. The docking procedure was centered on Glu22 and Asp23, using a grid box 60 × 60 × 60 Å, with the grid points separated by 0.375 Å. All protein visualizations were performed with PyMol viewer [30]. The lowest energy cluster for each ligand was subjected to further free energy (ΔG) and binding geometry analyses, as previously reported [28].

### Evaluation of Aβ_1–42_ aggregation by ThT fluorescence

Once the docking studies yielded results that allowed us to select four compounds for experimental studies, we evaluated their effect on Aβ_1–42_ pathological aggregation. For this purpose, lyophilized wild-type human Aβ_1–42_ peptide (chloride salt) was purchased from Calbiochem (Mexico). HEPES sodium salt (>99.5% purity) and ThT were obtained from Sigma–Aldrich (Mexico). A freshly prepared Aβ_1–42_ solution (50 μM in fresh MilliQ water) was incubated alone and in the presence of the selected compounds (100 μM) [26]. The samples were incubated at 37°C in a 0.5-cm path length quartz cell and stirred at 250 rpm. The increase in ThT fluorescence [31] was measured using a Perkin–Elmer LS-55 fluorescence spectrophotometer equipped with a water-jacketed cell holder for temperature control. The emission and excitation wavelengths were 445 and 480 nm, respectively. The fluorescence emission recordings were made using path-length quartz cuvettes. The buffer solution was 20 mM HEPES and 100 mM NaCl, pH 7.4, containing 3.3 μM ThT [26]. The inhibition of Aβ_1–42_ pathological aggregation for compounds 5, 8, 14 and 19 was calculated after 24 h of incubation.

### CD measurements

The lyophilized wild-type human Aβ_1–42_ peptide was diluted in MilliQ water to a final concentration of 50 μM, as reported previously [11]. The Aβ_1–42_ solution was incubated at 37°C in the absence and presence of the target compounds. CD spectra were acquired using a JASCO J-815 spectropolarimeter (Jasco, Easton, MD, USA) equipped with a PFD-425S Peltier-type cell holder for temperature control at 37°C and magnetic stirring. The CD spectra were recorded from 180 to 250 nm using 1.0-mm path-length quartz cells. The data were collected after a 3 h incubation because the principal conformational changes of Aβ_1–42_ have been found at this incubation time [26]. The data were corrected by subtracting the spectra of a sample that contained all of the components except Aβ_1–42_. The data were converted to mean residue ellipticity and analyzed using the Selcon [32] and K2D programs [33] to calculate the predominance of secondary structures.

### AFM

For visualization of Aβ_1–42_ alone or in presence of the compounds, 5 μl of a 50 μM Aβ_1–42_ peptide solution in the absence or presence of the selected compounds (100 μM) was incubated for 24 h, deposited onto glass slides and dried.

To determine whether the incubation of Aβ_1–42_ (50 μM) in presence of the best compound (100 μM) favors the formation of oligomers, 5 μL samples at 0, 1, 5 and 17 h of incubation at 37°C with shaking were collected, deposited onto glass slides and dried. In addition, samples of Aβ_1–42_ alone at the same incubation times were obtained to compare the results. All of the images were scanned in air by AFM (MultiMode V; Veeco, USA) using the tapping mode.

### Determination of IC_50_ of Aβ_1–42_ fibrillation by ThT fluorescence

Once the Aβ_1–42_ aggregation inhibitor activities were corroborated, the IC_50_ for the best compounds were determined as described below. After 24 h of incubation of Aβ_1–42_ (50 μM) with one of several concentrations of the selected compounds (0.001, 0.1, 1, 10, and 100 μM), the inhibition of Aβ_1–42_ fibrillization was measured using the ThT assay, as described above.

### Frontier orbitals.

Additional *in silico studies* were performed to determine the molecular basis of the recognition of Aβ_1–42_ by the best compounds because these studies provide electronic details regarding the compounds’ effects on Aβ_1–42_ conformational changes. The geometry of the ligands, the Aβ_1–42_ peptide and the ligand–Aβ_1–42_ complexes were fully optimized using the AM1 all-valence electron self-consistent field molecular orbital approximation [34]. This method is included in the Gaussian 2009 package of programs [35] with default parameters.

The HOMO, SOMO and LUMO energies [36] were determined for the structures obtained after interacting with the **Aβ-α**, **Aβ-RC** and **Aβ-β** peptides to understand their interaction with the peptide site. Molecular electrostatic potential maps (MEPs) [37] were obtained for the ligands to complete the electronic analysis. All of the geometric and electronic calculations were performed using the Gaussian 09 package of programs [36].

## Results

### Ligand selection

Most of the ligands that inhibit Aβ_1–42_ oligomerization had an amine, which is capable of acquiring a positive charge at pH 7.4, and an aromatic ring [10–12]. Therefore, we chose 26 compounds from the Sigma–Aldrich database (http://www.sigmaaldrich.com) with these chemical characteristics (Fig 1). ACh, which contains a quaternary amine, was selected as a reference ligand to evaluate the influence of the lack of an aromatic group on the recognition on Aβ_1–42_ (Fig 1; compound 26). The protonation states (pKa values) of the amine in the compounds were considered based on the ionizable groups at physiological pH.

### Docking studies

We employed three Aβ_1–42_ conformations to perform docking studies. The first conformer corresponded to the 1Z0Q structure obtained from the PDB, which corresponds to the classical α-helical conformation of Aβ_1–42_. According to the stride server, 43% of the residues were in the α-helix conformation. This conformer was labeled **Aβ-α**. The second conformer corresponded to the Aβ_1–42_ conformation obtained at 10 ns in the MD simulations, as reported in a previous work [26]. This conformer represents the RC, according to the determination of its secondary structure by the stride server (**Aβ-RC**; 90% random coil and 10% turn). The last conformer corresponded to an Aβ_1–42_ monomer from the 2BEG structure obtained from the PDB, which has a strand-loop-strand structure similar to what is observed in the mature Aβ_1–42_ fibrils. This conformer was labeled **Aβ-β** (43% β-sheet, 19% turn). The Aβ_1–42_ structures employed were chosen because the principal conformation of Aβ_1–42_ in the membrane is the α-helix [38]. However, in solution, Aβ_1–42_ can adopt different conformations, such as RC, a β-strand structure, and stable turns and bends. In addition, there are several reported conformational changes that occur during aggregation. Both the random coil to β-sheet and α-helix to β-sheet transitions occurred during Aβ folding and assembly. Importantly, the α-helix to β-strand transitions play a prominent role in the fibril assembly process [38].

The twenty-six ligands could be grouped into three families according to their chemical structures. The first group of ligands possessed an amine group that can acquire a positive charge at pH 7.4 and is capable of forming electrostatic interactions with Glu22 and Asp23 (Fig 1; compounds 1, 3–8, 11, 12, 17, 20 and 23–25). The second group consisted of molecules with an amino group covalently coupled to an aromatic ring (Fig 1; compounds 16, 18 and 19). The third group consisted of compounds that had an amine as part of their amide group (Fig 1; compounds 2, 9, 10, 13–15, 21 and 22). These amine groups are capable of forming hydrogen bonds with Glu22 and Asp23. In addition, we included ACh (Fig 1; compound 26) in the *in silico* studies to determine its selectivity for **Aβ-α**, **Aβ-RC** and **Aβ-β** because ACh contained a quaternary amine without an aromatic ring.

Compounds in the first group acted as Lewis bases, which could be protonated at physiological pH [39]. These docking results showed that the compounds formed electrostatic interactions with the carboxylate group of Glu22 and/or Asp23 residues of **Aβ-α** with high affinity and consequently showed lower ΔG values (Table 1) than **Aβ-RC** and **Aβ-β**. However, according to the theoretical docking studies, this was not the rule in all of the ligands tested.

**Table 1:** Amino acid residues of Aβ_1–42_ that interact with the compounds and their ΔG values obtained via docking studies.

Ligand	Aβ-α		Aβ-RC		Aβ-β	
	ΔG (Kcal/mol)	a.a	ΔG (Kcal/mol)	a.a	ΔG (Kcal/mol)	a.a
**1**	-5.5	Asp23, Ser26, Asn27	-4.5	Lys28, Gly29, Ala30, Ile31	-3.6	Lys28, Gly29, Ala30, Ile31
**2**	-4.4	Lys16, Phe20, Asp23, Ile31, Leu34, Met34	-4.5	Gly29, Ala30, Ile31, Ile32	-3.6	Gly29, Ala30, Ile31, Ile32
**3**	-5.4	Phe19, Asp23, Val24, Lys28, Leu34	-4.5	Phe19, Asp23, Val24, Lys28, Leu34	-3.4	Phe19, Asp23, Val24, Lys28, Leu34
**4**	-5.8	Gln15, Lys16, Phe19, Asp23	-4.4	Val18, Asp23, Gly25	-3.7	Val18, Asp23, Gly25
**5**	-6.2	Lys16, Phe19, Phe20, Glu22, Asp23, Asn27	-4.4	Val18, Phe19, Glu22, Asp23	-3.6	Ala21, Gly29, Ala30
**6**	-5.3	Phe19, Phe20, Asp23, Ile31, Ile34, Ile35	-4.8	Phe19, Glu22, Asp23	-3.7	Phe19, Glu22, Asp23
**7**	-5.9	Gln15, Lys16, Phe19, Asp23, Asn27	-4.6	Tyr10, Gln15, Phe20, Met35,	-3.7	Tyr10, Glu11, Gln15, Lys16, Phe20, Met35
**8**	-5.1	Phe19, Phe20, Asp23	-3.9	Phe19, Gllu22, Asp23	-3.6	Phe20, Gly29, Ala30, Ile31
**9**	-5.2	Asp23, Ile31	-4.1	Ser26, Ala30, Ile31, Ile32	-3.6	Ser26, Ala30, Ile31, Ile32
**10**	-4.0	Lys16, Ile31, Met35	-3.8	Ala21, Gly25, Ser26, Ala30, Ile31	-3.5	Ala21, Gly25, Ser26, Ala30, Ile31
**11**	-4.5	Lys16, Phe19, Phe20, Asp23, Ile31	-4.2	Phe19, Glu22, Asp23	-3.3	Phe19, Glu22, Asp23
**12**	-5.7	Phe20, Asp23, Ile31, Leu34	-4.5	Ala21, Gly25, Ala30, Ile31	-3.3	Ala21, Gly25, Ala30, Ile31,
**13**	-4.2	Phe19, Glu22, Asp23, Ser26, Asn27,	-4.3	Asp23, Ser26, Lys28,Gly29, Ile31	-3.4	Asp23, Ser26, Lys28, Gly29, Ile31
**14**	-5.0	Phe2, Phe19, Ile31	-3.9	Hys14, Phe19, Glu22, Asp23	-3.8	Gly29, Ala30, Ile31
**15**	-4.0	Phe19, Phe20, Gln15, Lys16, Asp23	-4.6	Ala21, Gly29, Ala30, Ile32	-4.2	Ala21, Gly29, Ala30, Ile32
**16**	-4.3	Val24, Lys28, Ile31, Ile32	-4	Lys16, Asp23, Gly25, Gly29	-3.1	Asp23, Gly25, Gly29, Ala30,
**17**	-4.1	Val24, Lys28, Ile31, Met35	-4.1	Val24, Ser26	-3.4	Val24, Ser26
**18**	-5.3	Val24, Lys28, Ile31, Phe20, Ile32	-4.1	Ala21, Gly25, Gly29, Ile31	-3.6	Ala21, Gly25, Gly29, Ile31
**19**	-4.1	Lys28, Ile31, Val24, Phe20	-4	Val18, Phe19, Glu22	-3.1	Phe20, Ala21, Leu34
**20**	-4.6	Phe19, Phe20, Asp23, Ile31, Leu34	-4	Hys14, Phe19, Glu22, Asp23	-3.3	Hys14, Phe19, Glu22, Asp23
**21**	-3.8	Asp23, Ile31, Met35	-4	Gly25, Gly29, Ala30, Ile31, Ile32	-3	Gly25, Gly29, Ala30, Ile31, Ile32
**22**	-3.7	Gln15, Phe19, Phe20, Asp23, Ile31	-4.5	Ala21, Gly29, Ala30, Ile31, Ile32	-3.7	Ala21, Gly29, Ala30, Ile31, Ile32
**23**	-5.3	Phe19, Phe20, Asp23, Asn27	-5	Asp23, Val24, Ser26	-3.1	Asp23, Val24, Ser26
**24**	-5.9	Phe19, Phe20, Asp23, Asn27,	-3.8	Ala21, Glu22, Ala30, Ile31	-3.2	Ala21, Glu22, Ala30, Ile31
**25**	-5.9	Lys16, Phe19, Phe20, Asp23	-4	Hys14, Val18, Phe19, Asp23	-3.7	Hys14, Val18, Phe19, Asp23
**26**	-3.6	Phe19, Ala21, Asp23	-4.3	Lys16, Leu17, Phe19	-4	Lys16, Leu17, Phe19

Although 60% of the selected molecules had these characteristics, the neighboring substituents of each compound modified the binding mode and consequently the ΔG values of Aβ_1–42._

**Fig 2:**
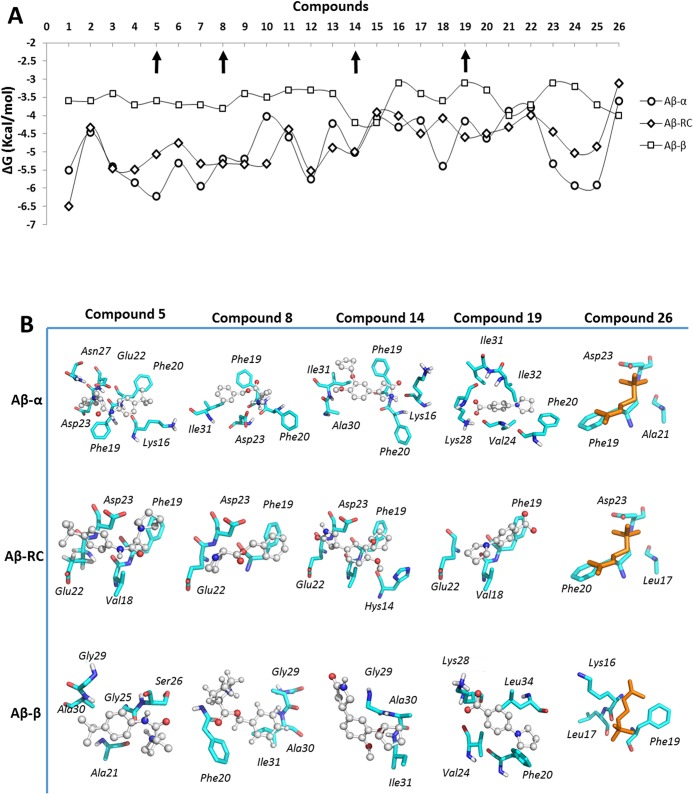
Docking results for the selected compounds with several Aβ_1–42_ conformers. ΔG values were obtained through docking studies of the ligands with **Aβ-α** (circles), **Aβ-RC** (rhombuses) and **Aβ-β** (squares) (A). The binding modes of compounds 5, 8, 14, 21 and 26 on **Aβ-α**, **Aβ-RC**, and **Aβ-β** (B).

According to the docking studies, all of the compounds generally showed less affinity to **Aβ-β** than to **Aβ-α** and **Aβ-RC** (Fig 2A), as has been recently reported [40]. In addition, despite several structural differences between the compounds, the ΔG values tended to be similar for all of the compounds obtained in the docking studies with **Aβ-β** and **Aβ-RC**, varying only slightly between the best and the worst compounds (1.0 Kcal/mol). Interestingly, the principal differences in the ΔG values were obtained from the docking studies with **Aβ-α**. Therefore, this result was employed as the starting point for choosing four ligands: one compound with the highest affinity for **Aβ-α** (compound 5); two compounds that showed similar affinities for **Aβ-α** and **Aβ-RC** (compounds 8 and 14); and one compound that showed higher affinity to **Aβ-RC** than **Aβ-α** (compound 19).

The docking analyses showed that the protonated amino group (tertiary amine) of compound 5 binds to the carboxylate groups of Glu22 and Asp23 of **Aβ-α** by electrostatic interactions, whereas the aromatic ring of compound 5 interacted with the lateral chain of Phe19 and Phe20 via π–π interactions. The tert-butyl group of compound 5 interacted with the methylene of Lys16 (Fig 2B) through hydrophobic interactions. Similarly, compound 5 bound to **Aβ-RC** via electrostatic interactions with Asp23 and Lys28, π-π interactions with Phe19 and hydrophobic interactions with Val18, as shown in Fig 2B. On the other hand, compound 5 interacts with Gly25, Ala21, Gly29 and Ala30 in **Aβ-β** via hydrophobic interactions, resulting in the highest ΔG value for this compound (Fig 2B).

Compounds 8 and 14 bound to both **Aβ-α** and **Aβ-RC** with similar ΔG values, though these values were lower for compound 8. The protonated amine of compound 8 interacted with the lateral chain of Asp23 via electrostatic interactions, and its aromatic ring formed π–π interactions with the lateral chains of Phe19 and Phe20 in **Aβ-α** (Fig 2B). Interestingly, compound 8 interacted with Asp23 and Glu22 on **Aβ-RC** via electrostatic interactions while maintaining π–π interactions with the lateral chain of Phe19, and the other interactions were lost (Fig 2B). The recognition of **Aβ-β** by compound 8 was preserved primarily by hydrophobic interactions with Gly29 and Ile31 (Fig 2B), and its aromatic ring interacted with Phe20 in the lateral chain. Compound 14 formed hydrophobic interactions with the lateral chain of Ile31 and Ala30 as well as π–π interactions with the lateral chains of Phe19 and Phe20 in **Aβ-α** (Fig 2B). These interactions were preserved between compound 14 and **Aβ-β** (Fig 2B). Compound 14 could establish hydrogen bond interactions with Glu22 and Asp23 on **Aβ-RC** as well as a π–π interaction with Phe19, as can be observed in Fig 2B.

Compound 19 lacked a protonated tertiary amine, forming only hydrogen bonds, similar to compound 14. Furthermore, it did not interact with the carboxylate groups of Glu22 and Asp23. However, the carboxylate group of compound 19 interacted with the lateral chain of residue Lys28 via electrostatic interactions, while its aromatic ring interacted with the lateral chain of residue Phe20 via π–π interactions and with the lateral chains of residues Val24, Ile31 and Ile32 in **Aβ-α** via hydrophobic interactions (Fig 2B). In contrast, compound 19 interacted with **Aβ-RC** via hydrophobic interactions with Val18, Phe19, and Glu22 (Fig 2B). Compound 19 interacted with the side chain of Phe20 through π–π interactions and with Leu34 and Val24 via hydrophobic interactions, while preserving the electrostatic interaction with Lys28 (Fig 2B).

For comparison, the quaternary amine of ACh interacted with the lateral chain of Asp23 via electrostatic interactions and formed hydrophobic interactions with Phe19 (Fig 2B). This binding mode was preserved in the interaction of ACh with **Aβ-RC**. ACh did not interact with Asp23 in **Aβ-β**, and the principal non-binding interactions established between ACh and **Aβ-β** were hydrophobic interactions with Leu17 and Phe19 (Fig 2B). The higher ΔG values exhibited by ACh led us to infer that the lack of an aromatic ring was responsible for these results, although the formation of electrostatic interactions between the tertiary amine of ACh and the lateral chain of Asp23 in **Aβ-α** were preserved.

### Evaluation of Aβ1–42 aggregation by ThT fluorescence.

**Fig 3:**
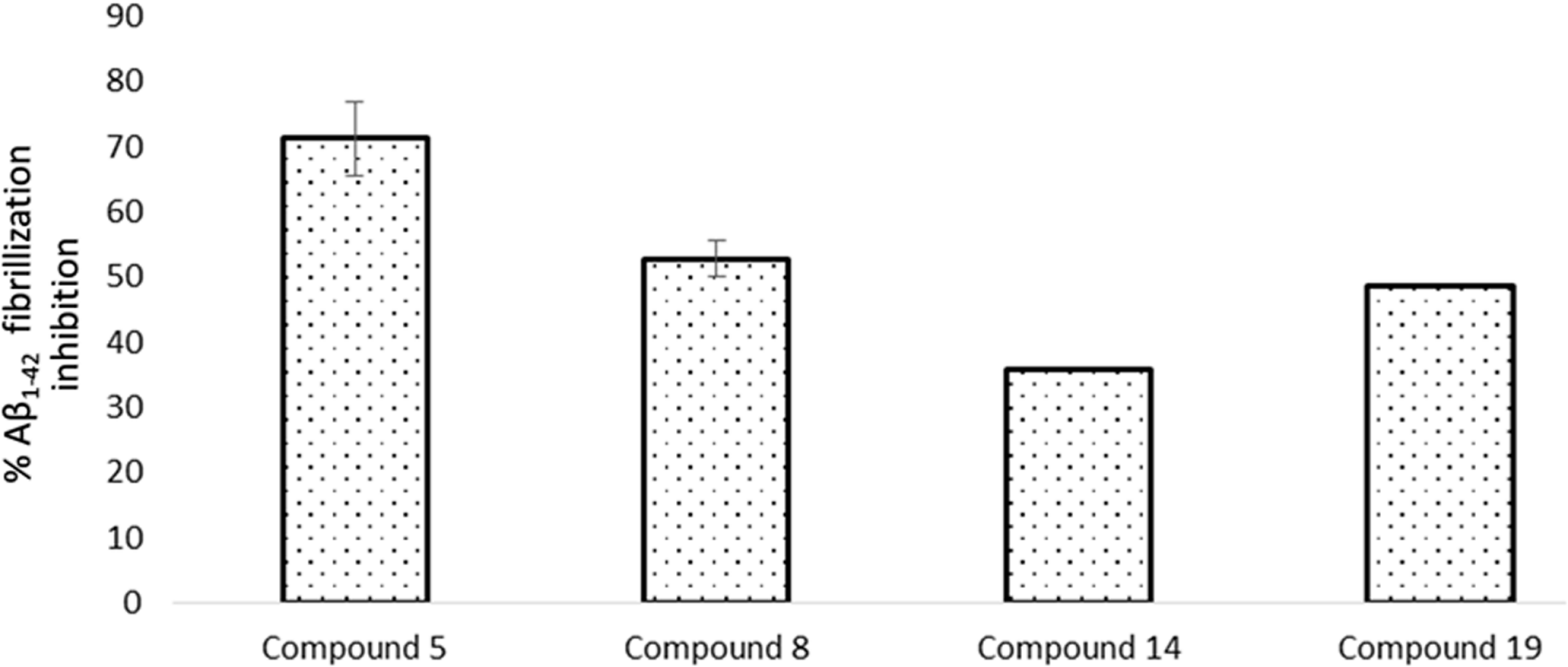
Results of the ThT fluorescence assay showing the effects of the selected compounds on the Aβ_1–42_ fibrillization process. Aβ_**1–42**_ (50 μM in MilliQ water) was incubated at 37°C in a quartz cell in the presence or absence of compounds 5, 8, 14 and 19 (100 μM) and stirred at 250 rpm for 24 h. The increase in ThT (3.3 μM) fluorescence was measured at the end of the incubation time.

*In vitro* studies were performed to determine whether the selected compounds could affect the pathological aggregation of Aβ_1–42_. For this purpose, a non-fibrillated Aβ_1–42_ peptide was the starting material used to monitor the fibrillization process [26]. The selected compounds inhibited the pathological aggregation of Aβ_1–42_, although compounds 5 and 8 were the best inhibitors. As shown in Fig 3, compound 5 inhibited the formation of Aβ_1–42_ fibrils by 73%, whereas compound 8 inhibited Aβ_1–42_ fibril formation by 53% at the end of the incubation period (24 h).

### CD

To determine the secondary structure of Aβ_1–42_ in the presence of the selected compounds, CD studies were performed. The spectra were corrected for the contribution of the CD spectra to the ellipticity of the compounds. After the samples were incubated without the compounds for 3 h under conditions that favored fibril formation, the CD spectra showed changes in the secondary structure, which represented the β-sheet (59.5%) conformation of Aβ_1–42_ (Table 2) [32]. When Aβ_1–42_ was incubated in the presence of compound 5, the CD spectra indicated a high proportion of RC content (56.5%), which resembles the conformation of unordered peptides [32]. A significant reduction in the proportion of β-sheet structure (15.5%) was also observed, though there was a small increase in the proportion of α-helix structure (18.5%), as shown in Table 2. Similarly, when Aβ_1–42_ was incubated in the presence of compound 8, the CD spectra indicated a high proportion of RC content (40.5%), though this was similar to the proportion of β-sheet structure (48%). A small increase in the proportion of α-helix structure (11.5%) was observed. For compounds 14 and 19, a high level of β-sheet content (52.5% for 14 and 52% for 19) was observed, although this proportion was lower than Aβ_1–42_ alone (59.5%).

**Table 2:** Proportions of Aβ_1–42_ secondary structures in the absence or presence of 100 μM of the selected compounds.

**Sample**	**α (%)**	**β-sheet (%)**	**RC (%)**	**Error**
**Aβ**_**1–42**_	2	59.5	38.5	11.5
**Aβ**_**1–42**_ **- compound 5**	18.5	15.5	56.5	11.5
**Aβ**_**1–42**_ **- compound 8**	11.5	48	40.5	6.5
**Aβ**_**1–42**_ **- compound 14**	14.5	52.5	33	11.5
**Aβ**_**1–42**_ **- compound 19**	5.5	52	42	11.5

### AFM

**Fig 4:**
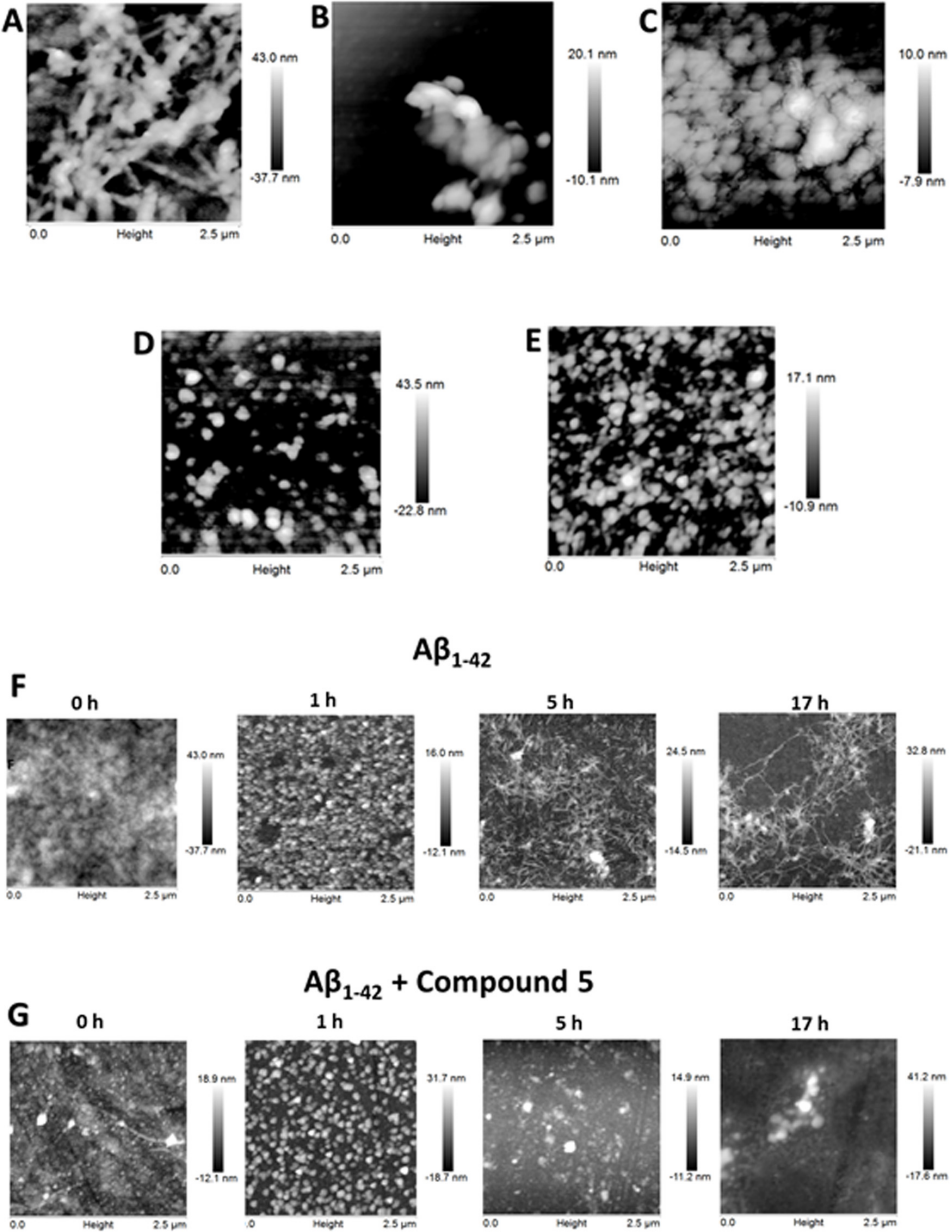
AFM analysis after incubating 50 μM Aβ_1–42_ alone or in the presence of the selected compounds at 100 μM after 24 h (A to E) or different incubation times (F and G). Aβ_**1–42**_ alone (A); Aβ_**1–42**_ and compound 5 (B); Aβ_**1–42**_ and compound 8 (C); Aβ_**1–42**_ and compound 14 (D); Aβ_**1–42**_ and compound 19 (E). Samples obtained at different incubation times for Aβ_**1–42**_ alone (F) or with compound 5 (G). Aβ_**1–42**_ (50 μM in MilliQ water) was incubated at 37°C in a quartz cell in the presence or absence of compounds 5, 8, 14 and 19 (100 μM) and stirred at 250 rpm for 24 h.

We used AFM to study the size and morphology of the Aβ_1–42_ aggregates after a 24 h incubation in the presence or absence of the selected compounds. The morphology of the Aβ_1–42_ aggregates in the presence of the selected compounds was different than the morphology of the fibrils obtained with Aβ_1–42_ alone. Fig 4A shows the characteristic fibrils of Aβ_1–42_, which have a width of ~30 nm and a height of ~20 nm. However, incubation of Aβ_1–42_ in the presence of compound 5 favored the formation of amorphous aggregates in which neither fibrils nor oligomeric species were found (Fig 4B).

In contrast, the incubation of Aβ_1–42_ with compounds 8 and 14 resulted in the formation of the characteristic spherical oligomers with a radius of 10–30 nm, as shown in Fig 4C and 4D, respectively. Fibrils were not observed. Additionally, as shown in Fig 4E, the incubation of Aβ_1–42_ with compound 19 resulted in the formation of a spherical species with a radius of 60–80 nm. However, these spheres were larger than the characteristic oligomers shown in Fig 4C and 4D.

To determine whether the incubation of Aβ_1–42_ and compound 5 favored the formation of oligomers prior to the formation of amorphous aggregates, several samples of Aβ_1–42_ alone and in the presence of compound 5 were obtained during different incubation times (0, 1, 5 and 17 h). No Aβ_1–42_ oligomer formation was observed (Fig 4F and 4G).

As shown in Fig 4F, the resuspension of Aβ_1–42_ results in a dense, homogeneous field of unaggregated peptide. In comparison, after 1 h of incubation, the height of the individual peptide structures as measured by AFM was 1.5 nm, which was in agreement with the expected size of a single Aβ_1–42_ monomer. Importantly, after 5 h of incubation, an increase in the formation of protofibrils (>200 nm) was observed. This was more evident after 17 h of incubation, with an increase in the presence of short fibrils (>500 nm).

In comparison, the presence of compound 5 did not alter the morphology of Aβ_1–42_ after 1 h of incubation, as shown in Fig 4G. However, after 5 h of incubation, the monomeric form of Aβ_1–42_ was maintained, and each individual peptide structure was 1.5 nm in height. However, the presence of small amorphous aggregates was observed, which was more evident after 17 h of incubation.

### Determination of IC_50_ of Aβ_1–42_ fibrillation by ThT fluorescence

Once compounds 5 and 8 were established as inhibitors of the pathological aggregation of Aβ_1–42_, their IC_50_ values were determined as 1.28 μM (compound 5) and 34.36 μM (compound 8) for use in future assays. Then, to determine how the binding modes of compounds 5 and 8 affected the pathological aggregation of Aβ_1–42_, the electronic behavior and the molecular basis of the recognition of Aβ_1–42_ by these compounds were analyzed using quantum chemistry.

### Frontier orbitals

**Fig 5:**
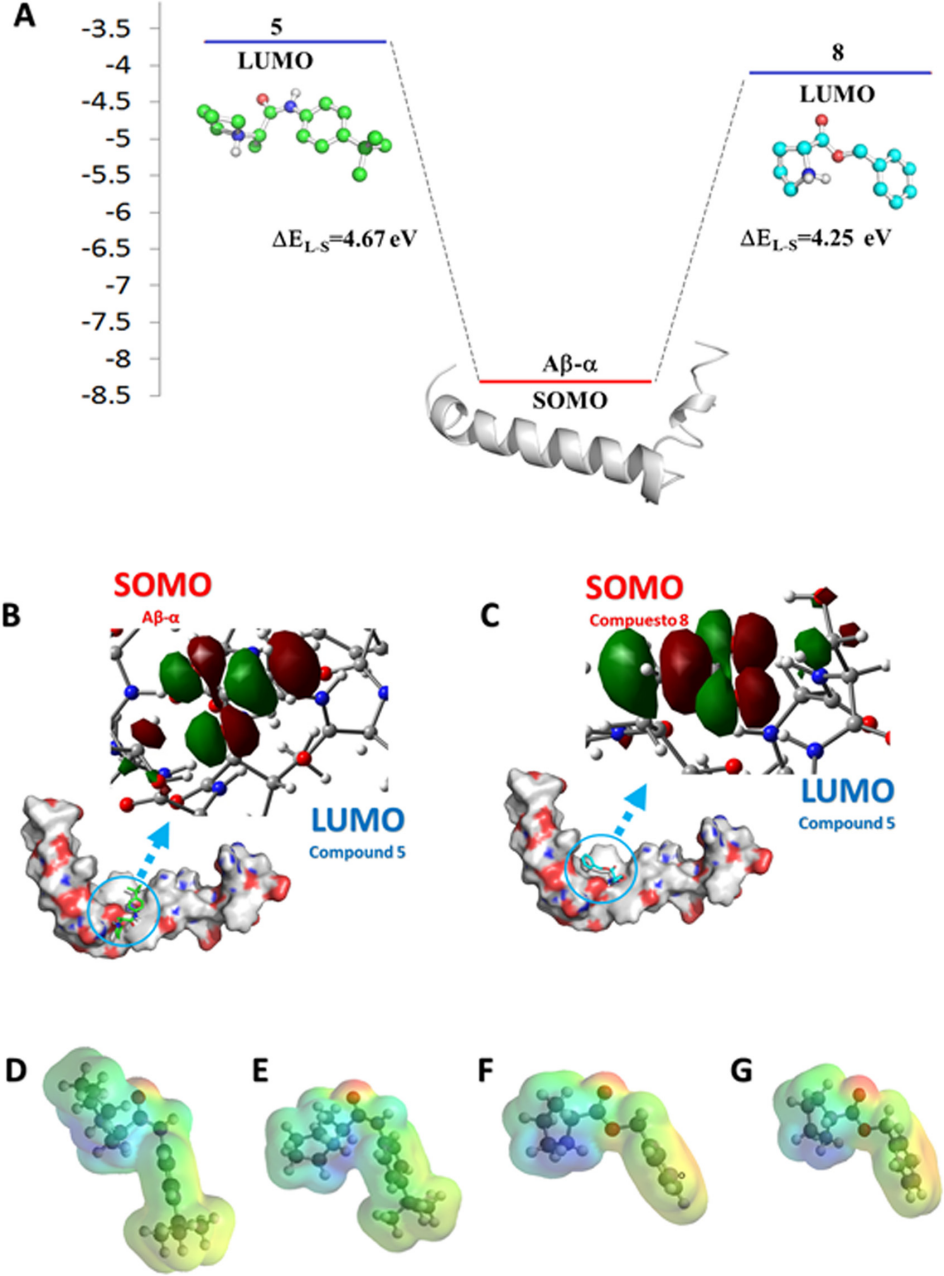
Spatial distribution of SOMO on Aβ-α. The illustration is based on the mapping of 0.032 isovalues and values onto a total electron density surface contoured at 0.0004 e/au3, which was based on AM1 semi-empirical calculations. The interaction between the LUMOs of compounds 5 and 8 and the SOMO of **Aβ-α**, in eV (A); **Aβ-α**–compound 5 complex SOMO (B); and **Aβ-α**–compound 8 complex SOMO (C) are shown. A map of the electrostatic potentials showing the most positive potential (deepest blue color) and the most negative potential (deepest red color) plotted on a surface with constant electron density (0.02 e/au3). MEP for compound 5 after docking studies with **Aβ-α** (D); MEP for compound 5 after docking studies with **Aβ-β** (E); MEP for compound 8 after docking studies with **Aβ-α** (F); and MEP for compound 8 after docking studies with **Aβ-β** (G).

A full geometry optimization was performed using the semi-empirical quantum mechanical program AM1 to calculate the heat of formation. The HOMO, SOMO and LUMO energies were also determined for the **Aβ-α**, **Aβ-RC** and **Aβ-β** conformations; for the compounds alone; and for the Aβ_1–42_–ligand complexes. Because the Aβ structure was used after the docking studies, it was not possible to obtain the HOMO energy from the presence of unpaired electrons that would indicate interactions with the ligand or whether the electrons were shared or transferred. The SOMO orbital energy of Aβ_1–42_ was obtained and was found to be more negative for **Aβ-α** (Table 3). Therefore, it was possible that the SOMO orbital energy of Aβ_1–42_ could have reacted with the LUMO energy of the ligands. Compounds 5 and 8 may act as inhibitors of Aβ_1–42_ oligomerization because their GAP values showed less of a difference in interaction energy between these two compounds and Aβ_1–42_ (Fig 5A). When the complex was formed between **Aβ-α** and compound 5, the LUMO orbital energy was located in the region where the ligand was recognized (Fig 5B), suggesting electronic interchanges.

**Table 3:** Comparison of LUMO, HOMO and SOMO (eV) and the electronic energies of the amino acid residues and compounds.

Structure	LUMO (eV)	HOMO (eV)	SOMO (eV)	Energy (ΔH), Kcal/mol
**Aβ-α**	-1.3		-8.35	-2309.8
**Aβ-RC**	-1.17		-7.56	-2176.3
**Aβ-β**	-0.96		-7.79	-2266.4
**Compound 5 (α)**	-3.68	-12.24		116.6
**Compound 8 (α)**	-4.1	-12.3		88.3
**Compound 5 (RC)**	-3.78	-12.66		117.21
**Compound 8 (RC)**	-4.1	-12.3		88.28
**Compound 5 (β)**	-3.83	-12.62		118.0
**Compound 8 (β)**	-4.1	-12.3		88.3

**(α)** After docking studies with **Aβ-α**

**(RC)** After docking studies with **Aβ-RC**

**(β)** After docking studies with **Aβ-β**

The recognition sites of compounds 5 and 8 corresponded to the region where the peptide turned to acquire a U shape (Ala21–Val24), and this was the location where the LUMO orbital energies for compounds 5 and 8 had more negative values (Table 3). Therefore, the interaction between compound 8 and **Aβ-α** was more favorable.

However, although compound 8 has the same recognition site for Aβ_1–42_ as compound 5, compound 8 did not prevent a conformational change in Aβ_1–42_. This could be due to the interaction maintained by each compound.

In addition, the interaction energy of compound 5 with **Aβ-α** (ΔE kcal/mol -50.29) was more stable than that of compound 8 (ΔE kcal/mol 3.49). This energy could explain why compound 5 had better ΔG values in the docking studies. Table 3 shows the ΔH values for compounds 5 and 8, which verified that the conformation of compound 5 alone was not the most stable, but it was stabilized when it complexed with Aβ_1–42_. This did not occur for compound 8.

To predict the behavior and reactivity of the molecules, the MEP was also obtained. MEP has largely been used as a molecular descriptor of chemical reactivity in many biological systems because it allows for visualization of the electrophilic and nucleophilic sites in a molecule [41]. In the MEP maps, there are three important colors that are used to indicate the value of the electrostatic potential: blue (positive), red (negative) and green (no charge). The surfaces with green colors indicate zero potential. As shown in Fig 3D–3G, compounds 5 and 8 had electronegative zones that were associated with the pair of electrons corresponding to the oxygen atoms. The positive electrostatic potentials were located at the acidic hydrogen atoms of the amine group.

Therefore, the GAP and MEP indicated that compounds 5 and 8 could be oligomerization inhibitors, but the interaction energy and conformational changes induced in **Aβ-α** by interaction with the ligands suggested that compound 5 was a better inhibitor than compound 8. Compound 5 exhibited different conformations when interacting with **Aβ-α** and **Aβ-β**, whereas compound 8 had the same conformation for both **Aβ-α** and **Aβ-β**. Compound 8 had more sites with free movement than compound 5 due to its bonds with sp^3^ hybridization, which could explain the conformations obtained in the docking experiments with compound 5. In particular, the hydrogen atom of the quaternary amine of compound 5 formed a double hydrogen bond with a carboxyl group of the peptide at distances of 2.17 and 1.87 Å, which indicated a strong interaction. Perhaps, this group could have indirectly encouraged the other interactions describing by the docking studies due to the volume of the isobutyl substituent.

Similarly, the interaction energy of compound 5 with **Aβ-RC** (ΔE kcal/mol -42.1) was more stable than that of compound 8 (ΔE kcal/mol -30.5). Table 3 shows the ΔH values for compounds 5 and 8, which verified that the conformation of compound 5 alone was not the most stable, but it was stabilized when it complexed with Aβ_1–42_. This did not occur for compound 8 and is similar to the behavior observed with **Aβ-α.**

In contrast, the interaction of compound 5 with **Aβ-β** showed a more extended conformation that did not provoke any conformational changes in Aβ_1–42._ A different conformation was obtained for each ligand. For instance, the interaction between **Aβ-α** or **Aβ-RC** and compound 5 favored the exposure of some atoms, such as the interaction between the hydrogen atom of the quaternary amine of the ligand and the oxygen atom of the carboxylic group of Aβ_1–42_. However, when compound 5 interacted with **Aβ-β**, its conformation was different, even though the distribution of the positive and negative electrostatic potential of the compound 5 was located in the same region of the molecule. According to the quantum results (AM1), the conformation of compound 5 in the interaction with **Aβ-β** was similar to that observed for compound 8 in both conformations of Aβ_1–42_. This showed the presence of intermolecular interactions via hydrogen bonding between the hydrogen atoms of the quaternary amino group and the amide or the carboxylic groups of the peptide.

## Discussion

The relationship between the pathological aggregation of Aβ_1–42_ and AD implies that the Aβ_1–42_ aggregation inhibitors should be able to slow disease progression [42–46]. The development of compounds with high affinity to the α-helix conformation could block the adoption of the β-sheet conformation by interacting with Asp23 or Lys28 and preventing the formation of the required salt bridge during the Aβ_1–42_ oligomerization process [21].

**Fig 6:**
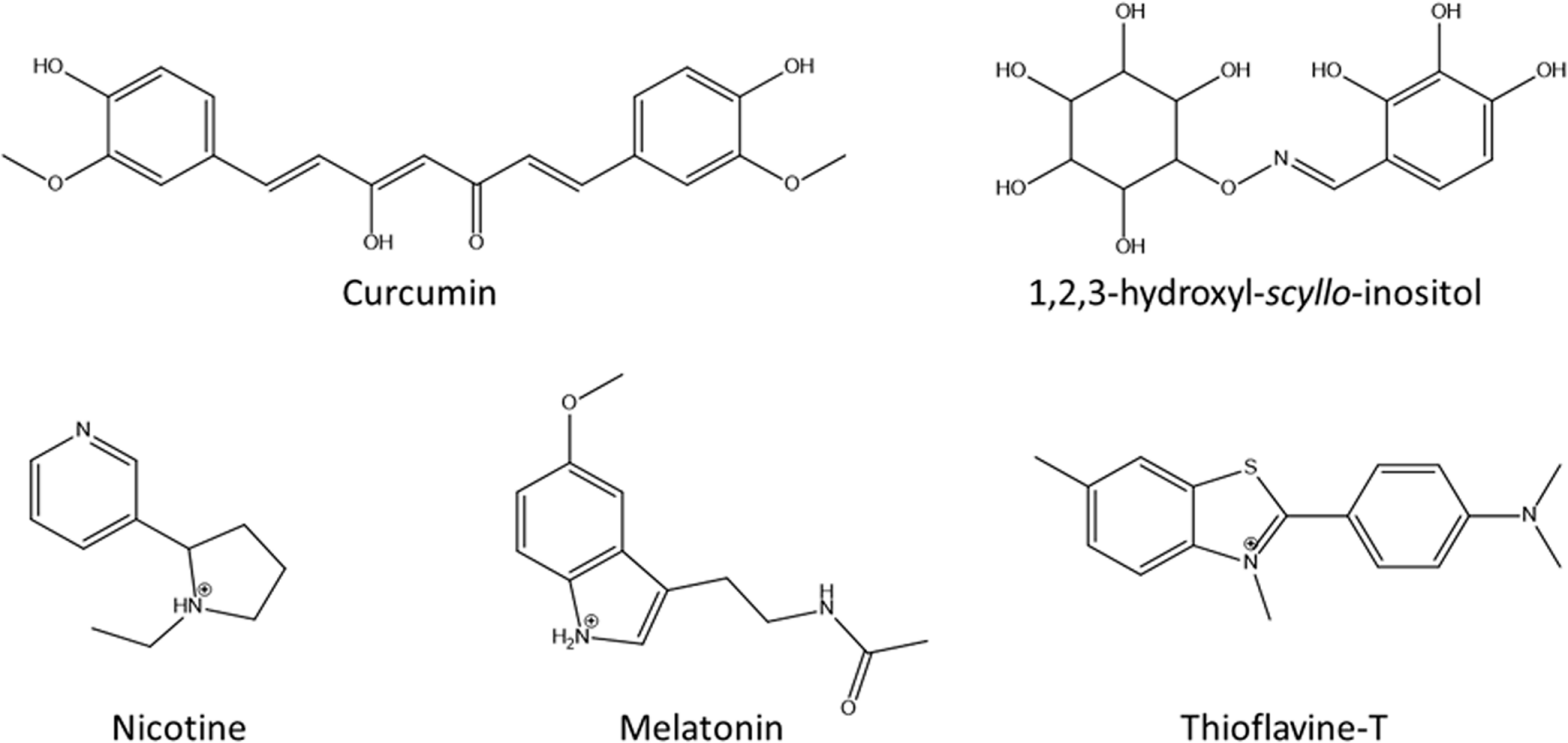
Chemical structures of the inhibitors of Aβ_1–42_ fibrillization. The majority of the compounds shared the presence of amines and/or aromatic rings.

Although a wide variety of organic compounds have been shown to inhibit Aβ_1–42_ aggregation (Fig 6) [10–15], many of them cannot be used because they are cytotoxic in cultured cells, favor the formation of oligomeric species, or have low bioavailability due to high molecular weights (>500 g/mol), which makes it difficult for them to cross lipid layers [42]. Because the cytotoxicity and genotoxicity of curcumin has been demonstrated in some cultured cells [47], several chemical analogs have been designed to overcome these limitations [12]. In addition, the activity of 1,2,3-hydroxyl-*scyllo-*inositol (Fig 6) as a fibril-forming inhibitor has been demonstrated by ThT fluorescence and AFM [15]. However, these compounds favor the formation of oligomeric species, which could result in toxic effects because of the correlation between the levels of soluble oligomers (rather than insoluble Aβ_1–42_ fibrils) and the extent of synaptic loss and cognitive impairment [48–51].

Melatonin and ThT have been shown be to inhibit Aβ_1–42_ fibril formation. These compounds share the presence of aromatic rings and protonated amines. These are important chemical characteristics for the recognition of Aβ_1–42_ because BTA-1, a neutral analog of ThT, is ineffective in inhibiting Aβ_1–42_ fibril formation [52].

Recently, NMR studies have demonstrated that 1,4-naphthoquinon-2-yl-L-tryptophan, which has been reported to reduce the level of aggregation and toxicity of Aβ_1−42_, could interact with Phe20, Ala21, Glu22, Val18 and Val24 [53, 54], demonstrating that the presence of a positive group and an aromatic ring are important for the recognition of the turn region in Aβ_1–42_.

The majority of the Aβ_1–42_ aggregation inhibitors mentioned above share tertiary amines and aromatic rings. This was taken into account when we selected 26 compounds with MW < 500 g/mol that each contain an amine group and an aromatic ring. After analysis of the *in silico* results, four ligands (compounds 5, 8, 14 and 19) were selected to evaluate their effects on Aβ_1–42_ oligomerization and the changes to its secondary structure. The four ligands included one with the highest affinity to **Aβ-α** (compound 5); two compounds that showed a similar affinity to **Aβ-α** and **Aβ-RC** (compounds 8 and 14); and one that showed a higher affinity to **Aβ-RC** than the **Aβ-α** conformation (compound 19).

The results of the *in silico* studies showed that compound 5 had the best affinity for **Aβ-α**, which explains its ability to inhibit the pathological aggregation of Aβ_1–42_. This resulted in the loss of ThT-positive Aβ_1–42_ β-sheets. The CD studies demonstrated the absence of the β-sheet conformation and the predominance of the RC structure, which indicates an unordered peptide conformation.

These results were mirrored by the AFM results, which showed structural changes in the Aβ_1–42_ deposits from the characteristic fibrils to amorphous precipitates when compound 5 was present (compound 5: Aβ_1–42_ ratio 2:1). This pattern of oligomerization inhibition is similar to that reported for copper [52]. By incubating Aβ_1–42_ in the presence of 200 μM copper under fibril-forming conditions, the appearance of fibrils decreased, and non-fibrillary, amorphous aggregates increased [52]. The formation of amorphous aggregates could represent a beneficial characteristic because recent evidence suggests that most of the detrimental forms of Aβ_1–42_ are soluble oligomers, whereas insoluble amorphous aggregates represent a less harmful, inactivated form of Aβ_1–42_ [51].

In contrast to the results mentioned above, although compound 8 had a positively charged group and an aromatic ring similar to compound 5, the absence of linearity did not provide a favorable arrangement of these functional groups. This structural difference completely changed the binding mode of compound 8. Nevertheless, the GAP results indicated that compound 8 could react better with Aβ_1–42_. In addition, the lack of hydrophobic substituents on the aromatic ring prevented the hydrophobic interactions with the methylene group of Lys16.

Although compounds 8 and 14 exhibited similar affinities for both conformations of Aβ_1–42_, these compounds were only capable of reducing the ThT fluorescence by 50%. These results are in agreement with the AFM observations, which showed the formation of the characteristic oligomer species that are related to neurotoxicity [9]. Although compound 14 established several hydrophobic and π–π interactions, the lack of ionizable groups diminished its ability to bind to homologous amino acids, resulting in its high binding energy.

The carboxylate group of compound 19 interacted with the side chain of Lys28, which may have prevented the formation of the salt bridge. However, the presence of the aromatic rings did not provide selectivity for **Aβ-α**, even though the turn portion (amino acid residues 27–30) showed fewer conformational changes during oligomerization in comparison with the β1 portion (amino acid residues 18–26) [22]. Compound 19 was only able to reduce ThT fluorescence for Aβ_1–42_, with similar proportions of β-sheet and RC conformations. The Aβ_1–42_ aggregates in the presence of compound 19 were similar to the spherical oligomer species produced by compounds 8 and 14, but with a higher ratio.

According to the results obtained above, oligomer formation can result when the compounds are able to bind the β-sheet and α-helix conformations with similar, rather than greater, ΔG values for Aβ_1–42_. Furthermore, because of the toxicity of these oligomers, it is necessary to prevent this event [10, 48].

Peptide structures that can interact with the negative region of Aβ_1–42_ (Glu22 and Asp23) have been proposed to be a useful alternative for the design of oligomerization inhibitors [55]. According to the results obtained, it can be noted that blocking the formation of the salt bridge only is insufficient to prevent Aβ_1–42_ oligomerization. In fact, several molecules have demonstrated this pattern of Aβ_1–42_ inhibition.

For these reasons, several chemical moieties are necessary for the preferential recognition of **Aβ-α** and must have a linear spatial arrangement that allows for interaction with residues 13–26, which are implicated in **Aβ-α** stabilization [20].

**Fig 7:**
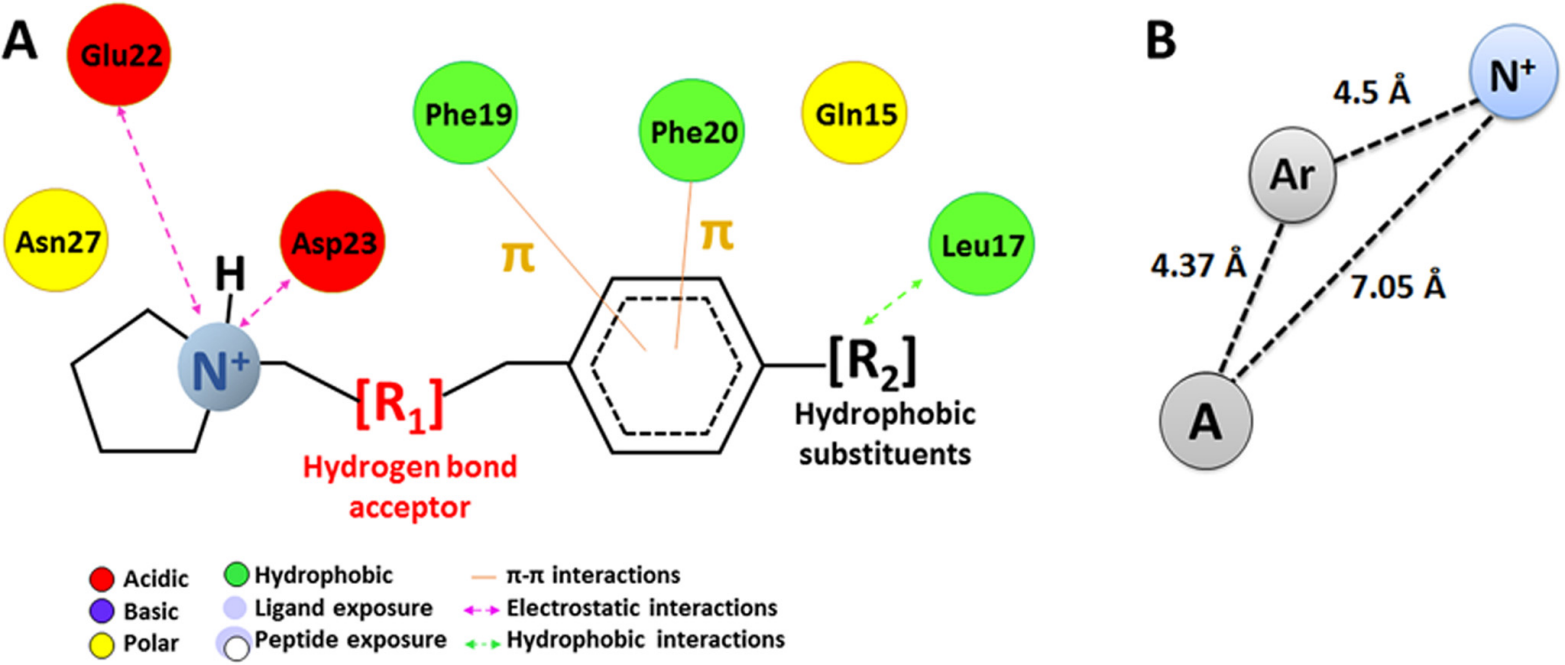
Proposed Aβ-α pharmacophore based on the studies with compound 5. Schematic representation of the polar and nonpolar interactions that favor the interactions with **Aβ-α** (A); distances between principal chemical groups, the protonated amine (N^+^), the aromatic ring (Ar), and Alkyl substituent (*Tert-B*) (B). The main interactions involved in the recognition of compound 5 are electrostatic interactions with Glu22 and Asp23, π-π with Phe19 and Phe20 and hydrophobic interactions with Leu17.

As shown in Fig 7A, the principal type of interactions that drive the selectivity of **Aβ-α** are electrostatic interactions with residues Glu22 and Asp23 and π–π interactions with Phe19 and Phe20. The formation of π–π interactions indicates the presence of an important moiety because the formation of strong π–π interactions between these chemical groups has been demonstrated to represent an intermediary stage in the unfolding process that drives the adoption of the β-sheet conformation [56].

The distance between both groups is important. Docking studies have shown that molecules with a distance of 4.3–5 Å between the amine and aromatic groups (Fig 7B) have selectivity for **Aβ-α** (4–7,11,12,24,25). However, the ΔG values vary considerably because the aromatic rings of the different compounds represent different substituents, and the presence of aliphatic substituents or aromatic rings favors the recognition of **Aβ-α**. The presence of polar or charged groups in the compounds increases the ΔG values, and consequently, the affinity. These characteristics are consistent with the design of curcumin derivatives, in which the presence of aryl rings with methoxy substituents are necessary for improving the inhibitory activity of curcumin [14].

Note that several drugs have these same characteristics, as was reported for melatonin [12]. Additionally, nicotine, a potent parasympathomimetic alkaloid, has shown the capability to retard amyloidosis by preventing α-helix to β-sheet conformational transformation, an event that is important in the pathogenesis of Alzheimer’s disease [11]. Moreover, nicotine has an aromatic ring and a tertiary amine that are capable of acquiring a positive charge at physiological pH.

The tricyclic antidepressant amitriptyline showed a significant potentiation of the non-toxic Aβ monomer with a concomitant decrease in the cytotoxic dimer Aβ load in triple transgenic mice [57]. This suggests that amitriptyline has a similar mechanism of action for Aβ_1–42_ aggregation inhibition. However, the multiple adverse effects of amitriptyline, such as nausea, psychosis, constipation, blurred vision, heart rhythm disorders, postural hypotension and extrapyramidal symptoms, prevent its long-term use. Galantamine, an AChE inhibitor, has shown concentration-dependent inhibition of both Aβ_1–40_ and Aβ_1–42_ aggregation [58].

**Fig 8:**
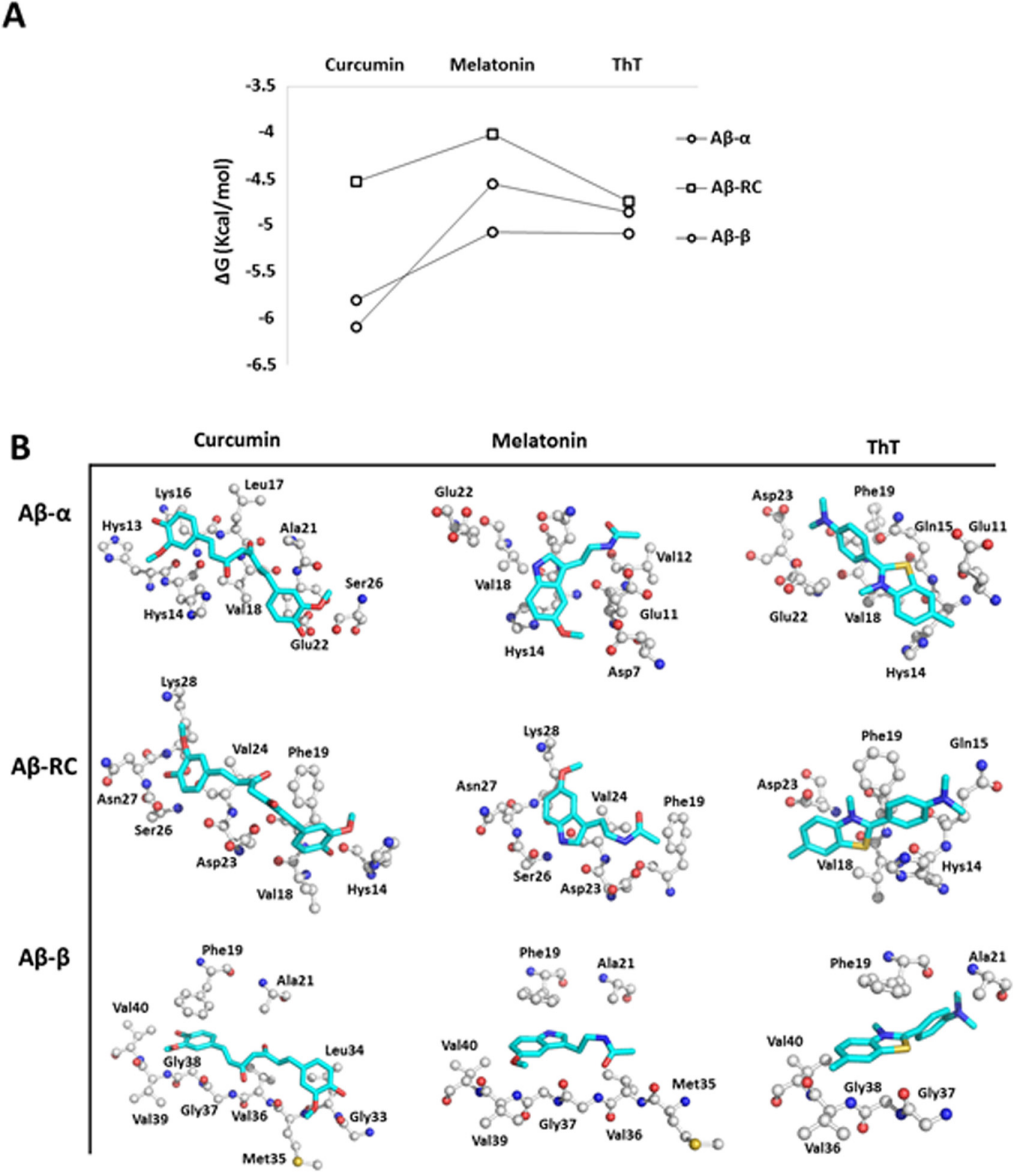
Docking results between curcumin, melatonin and ThT with several Aβ_1–42_ conformers. The methodology to obtain the complex is the same as mentioned above for the docking studies with the selected compounds. ΔG values were obtained through docking studies of the ligands with **Aβ-α** (circles), **Aβ-RC** (rhombuses) and **Aβ-β** (squares) (A). The binding modes of curcumin, melatonin and ThT on **Aβ-α**, **Aβ-RC**, and **Aβ-β** (B).

In addition, note that the compounds mentioned previously show similar interactions with the conformers of Aβ as compound 5. As shown in Fig 8, curcumin, melatonin and ThT exhibit higher affinity for **Aβ-α** and **Aβ-RC** than **Aβ-β** and importantly establish interactions with Glu22, Asp23, Phe19, Gln15, Lys17, Leu17 and Asn27 as shown for compound 5 on **Aβ-α** and **Aβ-RC**.

## Conclusion

These results show that compounds with different selectivities for the α-helix and β-sheet conformations of Aβ_1–42_ have different inhibitory effects on Aβ_1–42_ aggregation. Compounds with a higher affinity for **Aβ-α** could prevent the formation of the oligomeric species, whereas compounds with a higher affinity for the **Aβ-β** conformation allowed their formation. In particular, some chemical reactions were necessary for the preferential recognition of **Aβ-α**, including the establishment of electrostatic interactions with Glu22 and Asp23, π–π interactions with Phe19 and Phe20, and the presence of aliphatic substituents in the aromatic rings to establish hydrophobic interactions with the methylene side chain of Lys16. These findings allow for the identification of potential pharmacophores to inhibit Aβ_1–42_ oligomerization and to prevent the formation of oligomeric species.
